# Self-care Management Intervention in Heart Failure (SMART-HF): A Multicenter Randomized Controlled Trial

**DOI:** 10.1016/j.cardfail.2021.06.009

**Published:** 2021-06-20

**Authors:** Daniel Sahlin, Babak Rezanezad, Marie-Louise Edvinsson, Erasums Bachus, Olle Melander, Sofia Gerward

**Affiliations:** 1 Department of Emergency and Internal Medicine, Skane University Hospital, Skane, Sweden; 2 Department of Clinical Sciences, Lund University, Lund, Sweden; 3 Department of Clinical Sciences, Lund University, Malmö, Sweden

## Abstract

**Background:**

Self-care behavior is important in avoiding hospitalization for patients with heart failure (HF) and refers to those activities performed with the intention of improving or restoring health and well-being, as well as treating or preventing disease. The purpose was to study the effects of a home-based mobile device on self-care behavior and hospitalizations in a representative HF-population.

**Methods and Results:**

SMART-HF is a randomized controlled multicenter clinical trial, where patients were randomized 1:1 to receive standard care (control group [CG]) or intervention with a home-based tool designed to enhance self-care behavior (intervention group [IG]) and followed for 240 days. The tool educates the patient about HF, monitors objective and subjective symptoms and adjusts loop diuretics. The primary outcome is self-care as measured by the European Heart Failure Self-care behavior scale and the secondary outcome is HF related inhospital days.

A total of 124 patients were recruited and 118 were included in the analyses (CG: *n* = 60, IG: *n* = 58). The mean age was 79 years, 39% were female, and 45% had an ejection fraction of less than 40%. Self-care was significantly improved in the IG compared to the CG (median (interquartile range) (21.5 [13.25; 28] vs 26 [18; 29.75], *p* = 0.014). Patients in the IG spent significantly less time in the hospital admitted for HF (2.2 days less, relative risk 0.48, 95% confidence interval 0.32–0.74, *P* = .001).

**Conclusions:**

The device significantly improved self-care behavior and reduced in-hospital days in a relevant HF population. *(J Cardiac Fail 2022;28:3–12)*

In the context of heart failure (HF), self-care refers to activities performed with the intention of improving or restoring health and well-being, as well as treating or preventing the disease.^1^ It is widely understood that about 50% of HF hospitalizations are a direct consequence of inadequate self-care behavior.^2,3^ Trying to find means of enhancing the self-care behavior of patients with HF to decrease hospitalizations, is likely well worth the effort. Multidisciplinary HF clinics, including specialized HF nurses where both titrating patients toward target doses according to guidelines, but also striving to improve patients’ self-care behavior, have shown significant impacts on clinical outcomes.^4,5^ Owing to its resource efficiency relative to outpatient HF clinics, digital care, and more specifically medical and public health practice supported by mobile devices (mHealth),^6^ could have an important role in enhancing self-care behavior in this population.^7^

Despite having great potential, research in the field of mHealth and HF has generally not been performed on patients that represent the general Swedish HF population, thus making the generalizability of the findings low. A recent systematic review of mHealth HF research including more than 1700 patients, report the mean age in published studies as 61 years.^8^ Furthermore, 100% of the patients included, where the ejection fraction (EF) was reported, had HF with reduced EF (HFrEF), that is, an EF of less than 40% and more than 81% of the participating patients were male. This finding is in contrast with a European HF population where the mean age of the patients with HF is 77 years,^9,10^ HF with preserved EF accounts for 50% of the HF population,^11^and approximately 50% of the patients with HF are female.^10^ The European Society of Cardiology has put together an e-cardiology working group, which recently issued a position paper on challenges in digital health implementation in cardiovascular medicine, where they emphasize that any new e-solution must be designed for the population in question.^12^

The purpose of this current study, called “Selfcare Management Intervention in Heart Failure” (SMART-HF), was to evaluate if a novel home-based mHealth tool could improve self-care behavior and decrease HF-related in-hospital days in a population with more resemblance to the general Swedish HF population, compared with previous research. This work will allow for a better assessment of the tool’s suitability for implementation into clinical practice.

## Methods

### Study Design and Population

SMART-HF was a multicenter, randomized controlled clinical trial, recruiting patients with HF from 5 primary care centers and 2 hospitals in southern Sweden in the county council Region of Skåne with a follow-up of 8 months.

The study was approved by the regional ethics committee at Lund University, Sweden (protocol 2018/1, application nr 2017/956) and the research conducted conforms with the ethical principles of the Declaration of Helsinki. The study was registered at ClinicalTrials.gov with identifier: NCT03484286 before patient enrolment began.

Patients were eligible for inclusion if they had a diagnosis of HF according to the European Society of Cardiology guidelines,^13^ filled out the consent form, were considered able to handle the intervention by the recruiting health care professional and had at least 1 acute hospital admission for HF within the past 12 months or according to the recruiting health care professional after clinical assessment in risk of readmission. The clinical assessment was based on medical history, New York Heart Association (NYHA) functional classification and levels of N-terminal pro-B-type natriuretic peptide.

All recruited patients were listed at nurse-led outpatient HF clinics and all recruiting centers were connected to the Swedish Heart Failure Registry (SwedeHF, https://www.ucr.uu.se/rikssvikt-en/) assuring a standard of care throughout Sweden. According to centers’ protocol, 3 visits are the minimum scheduled outpatient visits during the first year of HF diagnosis and after that yearly visits. If unstable and/or in need of titration of medicines the recommendation is HF nurse visits every 2–3 weeks until stabilized. Both the control group (CG) and the intervention group (IG) were followed at outpatient HF clinics receiving the same standard of care.

Patients were assessed for eligibility by an health care professional (physician or HF nurse) at the participating center, either during a hospital admission or during an outpatient visit to the HF clinic at the primary care center. The recruitment of patients was always made at an outpatient visit to ensure proper follow-up in both CG and the IG. After providing informed consent in writing, baseline data was collected at inclusion and after 8 months. Data collected were age, sex, weight, EF, NYHA functional classification, blood pressure, heart rate, etiology of HF, routine laboratory tests according to the guidelines, comorbidities, and drug therapy. The data were collected from the patient’s medical record, examination of the patient, and questions to the patient and noted in a research case report form. The patient also filled out questionnaires about selfcare behavior and self-assessment of NYHA functional classification in person at the visit at the time of enrolment and after 8 months. The EF was taken from the latest echocardiography examination available in the patients’ medical record. The research nurse provided sealed, opaque envelopes to each recruiting site. The responsible health care professional at each site would then select an envelope from the batch of envelopes allotted to the center in question and open it to see if the patient was to be included in the CG or the IG. The randomization was performed locally at each site, 1:1 and the data analyst was blinded to participants allocation. Patients randomized to the IG had the intervention installed in their homes within seven days after randomization.

### Intervention

The patients in the IG were equipped with the home-based tool OPTILOGG (CareLigo AB, Sweden), a CE-marked class 1m medical device, at the earliest possible opportunity after the randomization. The patient received a home visit by a non-health care professional technician who installed the tool and instructed the patient on how to use it. The tool is based on a tablet computer wirelessly connected to a weight scale and incorporates symptom monitoring, interactive education, and adjustment of loop diuretics in the patient’s home. When the tool is prescribed to a patient, the responsible physician inputs patient-specific parameters to guide the flexible loop diuretics regimen and after deployment it works as a closed-loop system. No monitoring activities by the health care professionals are required after deployment; however, the trends can be shared with the health care professional during a visit at the patient’s discretion. The patient is encouraged to use the tool daily, and daily use involves registering weight, getting today’s dose of loop diuretics, and a brief education about living with HF. Every 5 days, the patient is asked to assess his or her symptoms on an ordinal scale on the screen. The tool thus supports the patient in exercising self-care, addressing maintenance, monitoring, and management, the 3 main components of selfcare in the context of chronic illness.^14^ If the tool detects a deterioration in HF status, the patient is encouraged to contact his or her health care professional. The same telephone number that the IG patients were encouraged to call by the tool in case of deterioration in HF status was also provided to the CG patients. The company providing the technology provided technical support via telephone during office hours.

### Outcomes

The primary outcome was self-care behavior measured by the European Heart Failure Self-care behavior scale (EHFScB-9), a valid and reliable instrument.^15^ It consists of 9 questions, each with 5 possible answers on an ordinal scale. The output ranges from 9 to 45, where a lower score denotes better self-care behavior and decreases the risk of adverse clinical outcomes.^16^ The outcome was measured at baseline and after 240 days.

The secondary outcome was the number of in-hospital days owing to HF after 240 days of intervention.

Other outcome measures were event-free survival, defined as the composite end point of time to the first occurrence of HF-related emergency room visit, HF admission, or death, a recommended end point for clinical studies in HF,^17^ as well as unplanned hospital visits owing to HF after 240 days of intervention.

We also analyzed system adherence to the tool that was defined as the number of days the patient used the tool (reading information, reporting symptoms or weighing themselves), divided by the number of days the patient has been equipped with it.^15^ No adjustments were made if the patient was admitted to the hospital and therefore was not able to use the tool.

All hospitalizations and emergency room visits during the follow-up time were adjudicated as being HF related or not, based on the diagnosis code (International Classification of Diseases code 10 I.50) in the medical records.

### Sample Size and Statistical Analyses

The primary and secondary null hypotheses were that there is no difference in self-care behavior, or in-hospital days between the CG and the IG after 240 days, respectively. We used the method to estimate sample size proposed by Li et al.,^18^ which was also used in the study by Hovland-Tånneryd et al.^19^ Because the current study sought to recruit older and thereby likely sicker patients, we assumed a 15% higher event rate overall than in the paper by Hovland-Tånneryd et al., and an effect size of 40% because the duration of the current study was one-third longer than the previous studies. This process yielded a sample size equal to 58 + 58 patients to reject the primary and secondary null hypothesis with 80% power. With an estimated dropout rate of 5%, the target for enrolment was set at 124 patients.

Baseline characteristics were analyzed using the χ^2^ test and a t test for independent samples for categorical, and continuous and normally distributed variables respectively. The selfcare behavior (EHFScB-9 score), being ordinal and non-normally distributed data, was analyzed using the Mann–Whitney *U* test. In-hospital days and unplanned hospital visits were modelled as over dispersed count data and analyzed using negative binomial regression. Adjusted and unadjusted Cox proportional hazards modelling was used to analyze event-free survival. The covariates included in any adjusted models, are those covariates where a significant difference between the CG and IG was detected after randomization. The effect on adherence to the tool by different factors was analyzed with multiple linear regression. P values of less than .05 were considered statistically significant and all significance tests were 2 tailed. The analyses were performed in accordance with a modified intention-to-treat principle on the full analysis set, consisting of all randomized patients who gave consent and began their assigned care. Specifically, all patients in the IG equipped with the tool were included in the analysis, irrespective of whether they decided to return the tool or stop using it before the end of the 240-day follow-up period. All statistical calculations were performed in IBM SPSS Statistics 25.

## Results

### Patients and Baseline Characteristics

A total of 158 patients were assessed as eligible between April 3, 2018, and February 12, 2019, at the participating centers. Out of these, 124 accepted and gave written consent and proceeded to be randomized 1:1 to either the CG or the IG. The primary care centers recruited 73 of the patients and 51 were recruited at the hospitals.

A total of 6 patients was excluded from the analysis because they never started their assigned care. The excluded patients were similar in age and comorbidities profile as the remaining population at baseline. All excluded patients did, however, have an EF of more than 40% and all had a hypertensive etiology. The participant flow is illustrated in

**Fig. 1:**
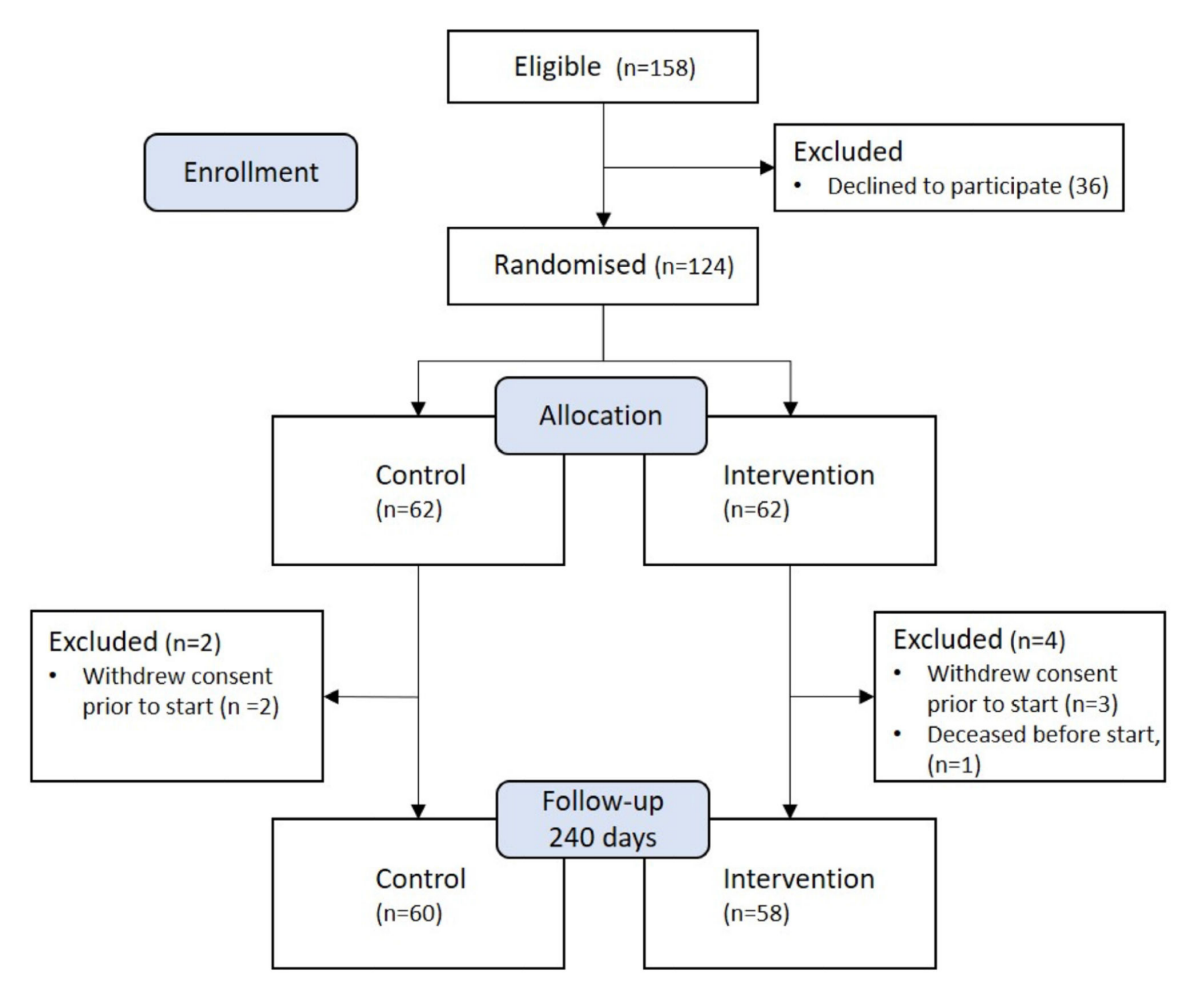
Participant flow.

Fig. 1. The analysis included 118 patients, with 60 in the CG and 58 in the IG. The patient characteristics are described in Table 1. The groups were well-balanced after randomization with the following exceptions: patients in the CG had a significantly lower prevalence of diabetes mellitus, significantly higher diastolic blood pressure, and better kidney function measured by estimated glomerular filtration rate (eGFR). The patients were on average 79 years old, 39% were female, and a majority of the patients had an EF of greater than 40% (i.e., HF with preserved EF or HF with midrange EF). The health care professional assessed that 29% of the patients were in NYHA functional class IIIa or higher, whereas self-assessed NYHA functional class resulted in that number being 47%.

### Outcomes

#### Self-care behavior

**Fig. 2:**
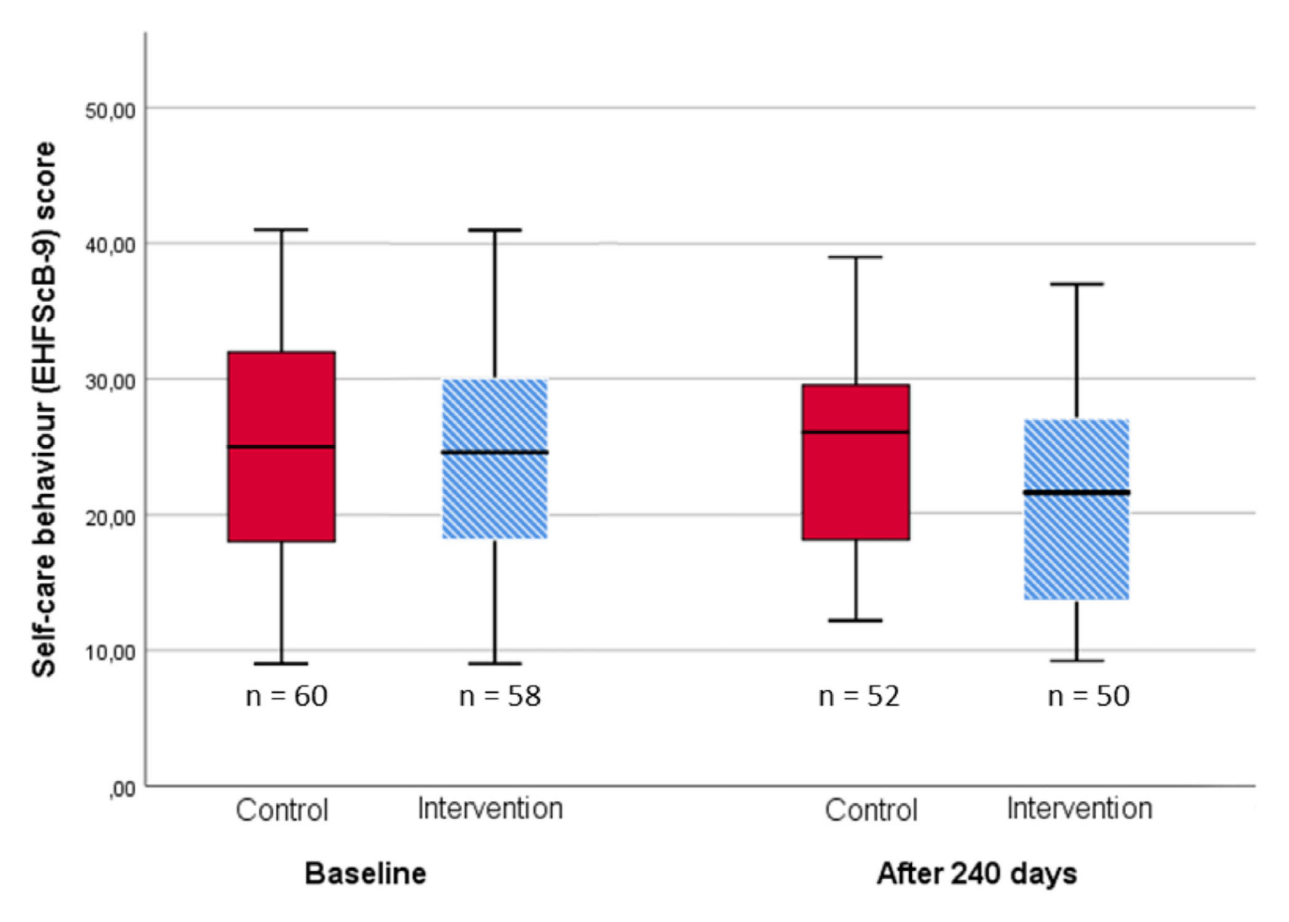
Self-care behavior at baseline and after 240 days of intervention. A lower European Heart Failure Self-care behaviour scale (EHFScB-9) score indicates a better self-care behavior. The error bars show the interquartile range.

At baseline, there was no difference in self-care behavior (reported as median EHFScB-9 [first quartile; third quartile]) between the groups (CG 25 [17.5; 32] and IG 24.5 [18; 30], *P* = .61 (*n* = 118). After 240 days, the IG had 21% (or 4.5 points) better self-care behavior (CG 26 [18; 29.75] and IG 21.5 [13.25; 28], *P*= .014) as shown in Fig. 2 (*n* = 102).

#### In-Hospital Days

After 240 days, a total of 754 all-cause in-hospital days were registered in 50 different patients, with 410 in the CG (6.8 per patient) and 344 in the IG (5.9 per patient) (relative risk [RR] 0.83, 95% confidence interval [CI] 0.56–1.22; *P* = .34). Of these in-hospital days, 367 (49%) were HF related, with 250 of these occurring for CG patients (4.2 per patient) and 117 for IG patients (2.0 per patient) (RR 0.48, 95% CI 0.32–0.74, *P* = .001, *n* = 118). The intergroup difference was significant after 30 days and persisted throughout the trial (Table 2).

Adjusting the analysis for age, sex, diabetes mellitus, diastolic blood pressure, and eGFR yields a RR of 0.35 (95% CI 0.21–0.57, *P*< .0001) after 240 days for HF-related hospital days, and for all-cause hospitalizations we obtain the adjusted RR was 0.50 (95% CI 0.31–0.81, *P* = .004).

#### Unplanned Hospital Visits and Event-Free Survival

There were a total of 131 hospital admissions, 40 emergency room visits without subsequent hospitalization, and 10 deaths recorded in the study (Table 3). Of these were 64 admissions and 13 emergency room visits related to HF, with 42 and 10 in the CG (0.87 per patient) and 22 and 3, respectively, in the IG (0.43 per patient), with a corresponding RR of 0.49 (95% CI 0.28–0.99, *P* = .044). The 10 deaths during the trial were distributed equally between the study arms.

**Fig. 3:**
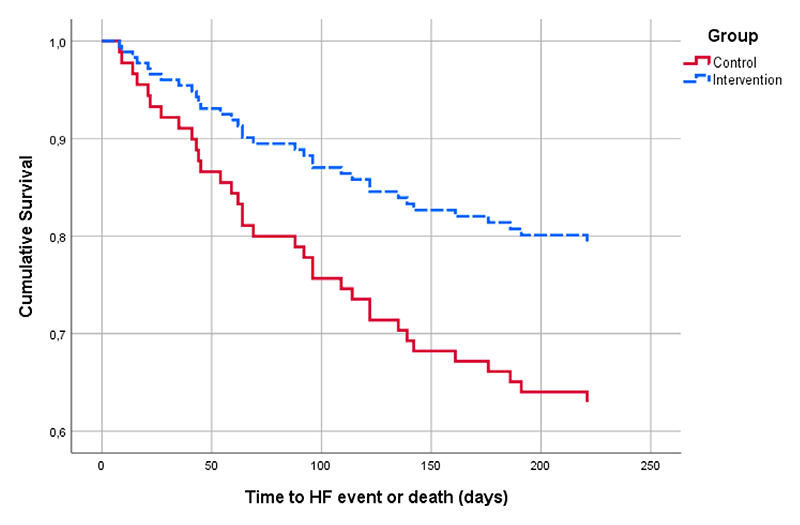
Survival functions for the control group (*solid*), the intervention group (*dashed*). HF, heart failure.

An unadjusted Cox regression was performed to analyze the HF-related event-free survival for the composite endpoint, yielding a hazard ratio of 0.50 (95% CI 0.24–0.98,P = .046) (Fig. 3). For all-cause hospital admission or death, the result was nonsignificant (hazard ratio 0.77, 95% CI 0.46–1.28, *P* = .32). Adjusting for sex, age, diastolic blood pressure, diabetes mellitus, and eGFR, we obtained a hazard ratio of 0.46 (95% CI 0.23–0.96, *P* = .033) for HF admissions, whereas the all-cause hospital admission or death analysis remained nonsignificant.

#### System Adherence and Data From the Tool

The median system adherence was 92% (interquartile range 67%–97%) the first 6 months and 85% (interquartile range 67%–95%) after 8 months. Sex, age, HF etiology, or disease state (NYHA functional class) did not predict system adherence.

There were in total 125 digital registered uptitrations in the mHealth tool in 38 different patients in the IG, and in 59% of these cases the weight returned to normal after the increased dose according to the digital registration.

No adverse events relating to the use of the tool were reported.

## Discussion

An 8-month intervention with the mHealth tool improved self-care behavior by 21% and decreased HF-related in-hospital days by 52%, corresponding with 2.2 days per patient. Furthermore, it led to a 50% decrease in the risk of an HF-related event or death.

The recruited patients from the southernmost part of Sweden are a good representation of patients with HF in terms of comorbidities, etiology, type of HF (i.e., HF with reduced EF, HF with mid-range ejection fraction, and HF with preserved EF), and age and sex distribution, both in the general Swedish population^9^ and for hospitalized patients,^20^ except for patients in NYHA functional class IV. All patients were followed up at nurse-led outpatient HF clinics and seem to be treated appropriately, with 86% on renin–angiotensin–aldosterone system blockade and 92% on beta-blockers.

The self-care behavior was improved by 4.5 points or 21%, which is similar to what has been published by others, where an intervention led to improvements by 3^2,21^ and 4^22^ points. However, greater improvements have also been published with 8 points improvement, including the previous studies of this mHealth tool.^19,23^ One of the reasons we did not see a larger improvement could be that the patients in this study had already received education at the HF clinics.

In the meta-analysis by Hovland-Tånneryd et al., where 2 studies of the same intervention as in our study were analyzed, they report 33% and 28% decreases in in-hospital days owing to HF after 6 months for the 2 included studies, respectively, and an adjusted total decrease based on analyzing both studies of 29.2% or 1.8 days per patient.^19^ The absolute decrease is similar to ours, but the relative decrease was larger in our material. This discrepancy might by explained by the fact that none of the patients in the study by Melin et al.^24^ had attended a HF clinic, and therefore may have been less optimally treated, with the result being more in-hospital days in their material, thereby decreasing the relative reduction.

An intervention consisting of a weight and symptom diary, flexible diuretics, and education sessions (i.e., very similar to the investigated intervention) by Cline et al. showed a 49% decrease in hospital days.^25^ The population investigated was similar to ours, in that the mean age was 76 and 47% were female.^25^ McAlister et al.^26^ published a meta-analysis of similar interventions, where they report a 43% decrease in HF hospitalizations based on data from 20 studies.

This study is the first on this mHealth tool in which a significant improvement in event-free survival as well as a significant decrease in unplanned hospital visits owing to HF is reported. It would be interesting to further explore and confirm these specific outcomes in future studies. The effects on the outcomes relating to hospitalizations and in-hospital care are likely the consequence of 3 different modes of action of the intervention. The first is the significantly improved self-care behavior, which leads to the patient needing less care.^27^ The second mode is that the flexible diuretics regimen can attenuate the effects of sudden weight gains, which in other RCTs has been shown to decrease hospitalizations by approximately 50%.^28,29^ It has previously been published that only a minority of patients adhere to weight based flexible diuretics, even after a recent hospitalization,^30^ whereas the investigated tool managed to return the weight back to normal after sudden weight gains in 59% of the cases in this study. The third mode is that, when the improved self-care behavior is not enough, the tool detects the deterioration in HF status and encourages the patient to seek care. This feature means that the patient will contact the health care professional at an earlier stage of deterioration, and a planned visit can be scheduled. Furthermore, it is common that patients misunderstand their drug regimens^31^ and, because the mHealth tool increases knowledge about HF,^24^ as well as significantly enhancing self-care behavior where drug compliance is 1 constituent, it is conceivable that the IG displayed improved adherence to pharmacologic therapy, which also should positively influence outcomes. This factor will be investigated further in future studies.

Last, adherence to the mHealth tool was an 85% median adherence after 8 months. The adherence at 6 months was 92%, which is similar to previous data indicating an adherence to the same tool of 94% at 6 months.^19^ These numbers are high, especially because health care professionals are not involved and do not actively encourage patients to use the tool once deployed. A qualitative study has reported that the investigated tool is easy to use and understand.^32^

The results from SMART-HF are very similar to previously published results from studies of the same mHealth tool. The population studied is a good representation of the general Swedish HF population, and it is reasonable to assume that these findings should translate well to a general setting of HF care.

### Methodologic Issues

The study size is limited, which should be considered when interpreting the results as the risk of a type I error increases with a small sample size. However, appropriate sample-size calculations were performed a priori. The results might add the most value when interpreted together with the other 2 studies investigating the same intervention.^19,24^

Owing to the nature of the intervention, placebo control was not possible. The mitigator for this was to provide all patients in the CG with the same contact phone number as the mHealth tool presents to patients in the IG and the instruction to call in case of worsening symptoms. Owing to 1 inclusion criterion being clinical assessment, it is possible that some healthier patients with HF with lower risk of admission were included in the study.

There were patients excluded from the analysis after randomization, and all had an EF of greater than 40% and a hypertensive HF etiology. However, the 2 study arms were still balanced with regard to these characteristics, and the excluded patients from the CG were not different from the patients excluded from the IG. We used the same approach to define the final analysis data set as in the TIM-HF2 study, published in The Lancet in 2018.^33^

Despite randomization, there was significantly worse kidney function (as measured by eGFR) and a higher prevalence of diabetes mellitus in the IG. The patients in the IG also reported more severe symptoms than what was assessed by the health care professional in terms of NYHA-class. These conditions (diabetes, worse kidney function and higher NYHA functional class) are the most common reasons of higher treatment costs for patients with HF.^34^ The IG was also 3 years older, although this difference was not significant (CG 77, IG 80, *P* = .13). These findings suggest that the IG patients were likely sicker and at a higher baseline treatment cost than the CG, but this only serves to decrease the probability of a type I error (false positive). That the IG patients were sicker, it is likely the explanation for why the reduction of all-cause in-hospital days was significant only in the adjusted analysis. Another potential bias is that social determinants play an substantial role in self-care behavior and the ability to take active part of one’s own health. In the context of a digital clinical trial, it may be possible to have biased population in regard to this factor.

Neither phone calls from patients to the health care professionals nor planned outpatient visits were analyzed and it is thus unknown if the mHealth tool affected the number of telephone and/or planned primary care contacts. A further limitation is that assessing a clinical meaningful minimal change is currently unclear and future studies will need to define this especially in the context of digital studies.

### Future Work

The effect of self-care on health care consumption among patients in NYHA functional class IV is not well-known, and a study recruiting patients with more advanced stages of HF would be of interest and needs to be studied further.

## Conclusions

The mHealth tool significantly increased self-care behavior, decreased HF-related in-hospital care, and unplanned hospital visits, as well as significantly increased event-free survival in patients who are a good representation of the general Swedish HF population. These effects were significant, despite all patients having been followed up at a nurse-led out-patient HF clinic.

## Figures and Tables

**Table 1:** Patient Demographics

		All (*N*= 118)	CG (*n* = 60)	IG (*n* = 58)
Age		79 ± 10	77 ± 11	80 ± 8
Female sex		47 (39)	28 (47)	19 (33)
NYHA functional class (health care professional assessed)				
	NYHA I	8 (7)	2 (3)	6 (11)
	NYHA II	75 (64)	39 (65)	36 (63)
	NYHA III	34 (29)	19 (32)	15 (26)
	NYHA IV	0 (0)	0 (0)	0 (0)
NYHA functional class (self-assessed)				
	NYHA I	4 (3)	4 (7)	0 (0)
	NYHA II	57 (49)	30 (51)	27 (47)
	NYHA III	51 (44)	24 (40)	27 (47)
	NYHA IV	4 (3)	1 (2)	3 (6)
HF type				
	HFrEF	53 (45)	27 (45)	26 (46)
	HFmrEF	30 (26)	13 (22)	17 (30)
	HFpEF	34 (29)	20 (33)	14 (25)
Etiology				
	IHD	47 (40)	21 (35)	26 (45)
	HTN	53 (45)	27 (45)	26 (45)
	CMP	8 (7)	2 (3)	6 (10)
	Other	21 (18)	14 (23)	7 (12)
Comorbidities				
	DM	46 (39)	18 (30)	28 (48)
	COPD	23 (19)	11 (18)	12 (21)
	HTN	60 (51)	29 (48)	31 (53)
	KD	26 (22)	15 (25)	11 (19)
	AF	72 (61)	37 (62)	35 (60)
Device (PM, ICD, CRT-P, CRT-D)		26 (22)	13 (22)	13 (22)
Hospitalizations past 12 months		1,2 ± 1,1	1,2 ± 0,92	1,2 ± 1,3
Blood pressure				
	Systolic	124 ± 20	124 ± 16	125 ± 23
	Diastolic	71 ± 12	73 ± 12	69 ± 11
Heart rate		71 ± 12	71 ± 11	71 ± 13
creatinine		125 ± 79	115 ± 63	134 ± 91
eGFR		45 ± 19	48 ± 21	42 ± 16
NT-proBNP		3265 ± 4951	3191 ± 4892	3339 ± 5008
Drug therapy				
	ACEi	62 (53)	32 (53)	30 (52)
	ARB	32 (27)	17 (28)	15 (26)
	RAS	92 (78)	47 (78)	45 (78)
	ARNI	7 (6)	4 (7)	3 (5)
	Beta blocker	109 (92)	56 (93)	53 (91)
	CCB	17 (14)	8 (13)	9 (16)
	Diuretics	105 (89)	53 (88)	52 (90)
	MRA	33 (28)	13 (22)	20 (34)

Values are *n* (%) or mean ± standard deviation. ACEi, angiotensin-converting-enzyme inhibitor; AF, atrial fibrillation; ARB, angiotensin II receptor blocker; ARNI, angiotensin receptor-neprilysin inhibitor; CCB, calcium channel blocker; CMP, cardiomyopathy; COPD, chronic obstructive pulmonary disease; CRT-D; CRT-P; DM, diabetes mellitus; eGFR, estimated glomerular filtration rate; HFmrEF, heart failure with midrange ejection fraction (40% ≤ EF ≤ 50%); HFpEF, heart failure with preserved ejection fraction (EF > 50%); HFrEF, heart failure with reduced ejection fraction (EF < 40%); HTN, hypertension; ICD, implantable cardioverter-defibrillator; IHD, ischemic heart disease; KD, kidney disease; MRA, mineralocorticoid receptor antagonist; NT-proBNP, N-terminal pro-B-type natriuretic peptide; NYHA, New York Heart Association; PM, pacemaker; RAS, renin–angiotensin system.

**Table 2:** Unadjusted and Adjusted Effects on HF-Related In-Hospital Days

Follow-up Time (Days)	HF-Related In-Hospital Days Per Patient		Rate-Ratio	*P* Value	Adjusted Rate Ratio	*P* Value (Adjusted)
	CG (*n* = 60)	IG (*n* = 58)				
30	0.37	0.05	0.14	.004	0.13	.005
60	1.0	0.31	0.31	.001	0.17	<.0001
90	1.73	0.55	0.32	<.0001	0.19	<.0001
120	1.85	0.86	0.47	.003	0.27	<.0001
150	2.15	1.17	0.55	.012	0.39	.001
180	2.67	1.40	0.52	.005	0.38	<.0001
210	3.37	1.83	0.54	.006	0.40	<.0001
240	4.17	2.02	0.48	.001	0.35	<.0001

CG, control group; IG, intervention group. Other abbreviations as in Table 1.

**Table 3:** Distribution of Events and Number of Patients

	CG				IG			
	No. of Events (Rate Per Patient)		No. of Patients (%)		No. of Events (Rate Per Patient)		No. of Patients (%)	
All-cause admissions	77 (1.3)	29 (48)	54 (0.93)	21 (36)
HF admissions	42 (0.70)	17 (28)	22 (0.38)	11 (19)
All-cause ER	23 (0.38)	14 (23)	17 (0.29)	14 (24)
HF ER	10 (0.17)	7 (12)	3 (0.05)	2 (3)
All-cause mortality	5 (0.08)	5 (8)	5 (0.09)	5 (9)

ER, emergency room. Other abbreviations as in Table 2.
